# Orthopedic treatment of children with scoliosis: what are the consequences on muscular force?

**DOI:** 10.1186/1748-7161-8-S1-O17

**Published:** 2013-06-03

**Authors:** I Courtois, C Morizot

**Affiliations:** 1 Unité Rachis, Hôpital Bellevue –CHU de Saint-Etienne, Saint-Etienne, France

## Background

We want to have a scientific answer to the question of many parents; does a brace affect the muscle structure of the trunk?

## Aim

We evaluated by isokineticism the development of muscular forces of the trunk, and the knee, in a group of adolescents undergoing orthopedic treatment for scoliosis; the study took place over 6 months

## Methods

The study included 18 teenagers, mean age of 12.75 +/- 1.66 affected by a progressed idiopathic scoliosis, with mean Cobb angle of 29 +/- 10 degrees at Risser 0 to 3.

A Cybex dynamometer was used to measure isokinetic trunk, and knee muscle strength, before the start of orthopedic treatment and 6 months after treatment commenced.

The isokinetic parameters measured were the maximum momentum for the flexor and extensor muscles, of the trunk, and knee, at different angular velocities, as well as the agonist/antagonist ratios. Seven healthy subjects of comparable age (12.57 +/- 1.27) served as a control group, evaluated just at the first stage of the study. The statistical analysis included averages, standard deviation, and student test.

## Results

In the follow-up, we observed a significant increase in both the height and weight of the scoliotic subjects, which shows they are growing. At the same time, Cobb angle has decreased a great deal. At the initial stage of analysis, before any treatment, all trunk parameter results for the scoliosis group are slightly lower than the control group, but the difference is not statistically significant. Moreover, there is a bigger gap between scoliosis and the control in extensor parameters than in flexor parameters. As far as the knee parameters, the comparisons between scoliosis and control groups are not conclusive. Whereas, comparisons between the pre-treatment stage and after 6 months, in scoliotic group, showed some increase but the different is not statistically significant. Comparisons between the pre-treatment stage and after 6 months in scoliotic group treated orthopedically showed a significant increase in strength of the flexor and extensor muscles of the trunk with a significant increase of the flexor- extensor ratio. So, the increase is more significant in flexor muscle than in extensor muscle. The main limits of this study were the small sample and the absence of a control adolescent group at 6 months, so that development of muscular strength in healthy teenagers is unknown.

## Conclusions

It can be concluded, however, from the results of this study that there is no negative effect on muscular strength in the trunk caused by the on-going orthopedic treatment of scoliotic growing adolescents. Moreover, this study shows us that extensor muscles seem to be more affected in scoliosis than controls, and also more affected by the orthopedic treatment. This should be taken into account in physiotherapy exercises.
